# Evaluating the use of AI in the design of learning situations by university students of early childhood education

**DOI:** 10.3389/fpsyg.2025.1604414

**Published:** 2025-09-24

**Authors:** Pilar Ester Mariñoso, Presentación Ángeles Caballero García, Isabel Morales Jareño, Emilio Cañadas Rodriguez

**Affiliations:** Facultad de Educación, Universidad Camilo José Cela, Spain

**Keywords:** artificial intelligence (AI), higher education (HE), mathematics education, academic performance, pedagogical innovation

## Abstract

The use of Artificial Intelligence (AI) in Higher Education (HE) is an expanding reality, exerting an increasingly significant influence on teaching and learning processes. Its integration into the university environment is reshaping how instructors design and deliver their courses while providing students with new opportunities to personalize their learning experiences. Not only does AI enable task automation and resource optimization, but it also presents methodological, technological, and ethical challenges that warrant thorough investigation. In this context, we assessed the academic performance of two hundred and thirty eight students enrolled in an undergraduate degree program for early years education teachers, defining performance as their ability to design learning situations aimed at promoting mathematical thinking in young children. Our analysis distinguished between those who used AI to complete the task and those who relied on traditional teaching resources. To this end, we adopted a sequential explanatory mixed-methods design. In the quantitative phase, we employed a quasi-experimental post-test only design with experimental and control groups. In the qualitative phase, we conducted an in-depth analysis of the learning materials produced by the students in the experimental group with the support of AI. The findings indicate that the application of AI brought about significant changes in the teaching-learning process. The experimental group obtained better academic results than the control group. These results underscore, on one hand, the transformative potential of AI to improve pedagogical practices, and on the other, the need for research to examine its long-term effects on student learning, teacher engagement and ethical use, and the development of HE curricula that include AI as a teaching resource.

## 1 Introduction

Artificial Intelligence (AI) is reshaping and redefining content creation by leveraging initial datasets to generate, optimize, and personalize educational materials more efficiently (Jiao, 2024; Navas-Franco et al., 2024). Not only does AI facilitate the automation of repetitive tasks and streamlining processes, but it also permits users to have more free time to focus on strategic and creative aspects of content development (Katiyar, 2024). However, AI also presents challenges that must be addressed from social, technological, scientific, and educational perspectives (Bit et al., 2024). In this respect, empirical evidence underscores AI's significant impact on education, enhancing teaching-learning practices, and improving student academic performance.

## 2 Theoretical framework

It has been 70 years since John McCarthy coined the term *Artificial Intelligence* (Cristianini, 2016), with a huge step forward over the past 7 years (Chu et al., 2022); and it is not until 2017 that academic discourse on the use of AI in HE has gained traction. Nowadays, AI's scope, meaning, and application have expanded significantly across all educational levels, and its use helps to personalize learning, guide and support individual students considering their own preferences or personal characteristics (Hwang, 2014; Hwang et al., 2020).

As noted by (Chan 2023), AI's accessibility and integration have permeated all educational domains and have also underscored the urgency for universities to develop AI-specific educational policies to equip students with the necessary skills to work with and understand this technology. Among those skills the mixture of ethical, technical, and human skills is necessary to prepare students to confidently face with the working future (Pervez et al., 2024; Qudrat-Ullah, 2025), and be aware of how to be responsible, effective and curious to inquire into innovative technologies that add value to any context. Moreover, universities bear the responsibility of preparing graduates to actively engage with AI-driven developments and emerging societal issues linked to their proliferation.

From a foundational perspective, AI is defined as the machines' capacity to imitate human intelligence (Turing, 1950), and over time, AI has evolved through different paradigms depending on its capabilities. Three primary AI waves can be identified: the first so-called symbolic AI operates based on predefined logic and can diagnose diseases; the second wave, known as data-driven AI, utilizes machine learning and data mining techniques; and the third one is referred to as context-aware AI, which is capable of understanding the real world and offering innovative solutions to novel or previously unknown problems (Holmes et al., 2019; Franganillo, 2023; Zhang et al., 2023). In any case, the current iteration of AI fosters uncertainty, particularly in education, where it aims to address complex objectives.

### 2.1 Evolution of AI in education

The trajectory of AI in education spans from the development of ELIZA in 1966 to the launch of ChatGPT in 2022 (Martins Ramos, 2023). While chatbots such as ALICE (1990's) improved response coherence without achieving contextual understanding, a significant transformation occurred with OpenAI's ChatGPT, a tool that takes advantage of deep neural networks and advanced machine learning models (Serrano and Moreno-García, 2024). The emergence of ChatGPT marks a turning point, and its potential long-term impact on education is projected to be substantial.

The present study focuses on generative AI, a subset of AI that has sparked considerable debate due to its increasing integration into educational environments (Franganillo, 2023). The integration of generative AI into educational systems has gained global relevance, particularly following the COVID-19 pandemic (Nedungadi et al., 2024). The limitations of traditional education models during the pandemic highlighted the need for AI-driven applications that could support students at risk of falling behind in fundamental skills such as those of literacy and arithmetic capabilities (Goessmann et al., 2023; Pantelimon et al., 2021; Slimi, 2023).

In this respect, UNESCO (2019) established three primary intersections between AI and education: learning with AI (using AI tools in classrooms), learning about AI (understanding AI technologies and methodologies), and preparing for AI (enabling all citizens to comprehend AI's potential impact on human life). These dimensions underscored the importance of equipping educational communities with the necessary competencies to critically and productively engage with AI technologies.

AI and education have converged in the realm of values, judgements and politics (Selwyn, 2019), and due to the significant role technology plays now more than in previous eras, the current generation is technologically literate (Limna et al., 2022). It is also relevant to note that contemporary education faces the challenge of developing innovative teaching and learning practices that uphold the principles of quality, inclusion, and equity, aligning with the Sustainable Development Goal 4 (SDG4) of the 2030 Agenda (García-Martínez et al., 2023; Jiao, 2024).

### 2.2 Artificial intelligence (AI) in Higher Education (HE): opportunities and challenges

The impact of AI in education was unknown or even disappointing, due to the limited progress achieved (Bates et al., 2020; Serrano and Moreno-García, 2024), and as (Zawacki-Richter et al. 2019) found out in their review, the low participation of education professionals shows it is necessary to focus on the pedagogical and ethic aspects in the application of AI in educational contexts. Accordingly, in the university sphere, AI presents both opportunities and concerns. A major issue is students' potential over-reliance on AI tools, which may undermine critical thinking and original writing skills (Chan, 2023; Civil, 2023; Warschauer et al., 2023). In the United States, a 2023 survey cited by Chan revealed that nearly one-third of students (out of 1,000 respondents) used AI chatbots like ChatGPT for written assignments, with 60% relying on AI for more than half of their tasks. While students were aware of the ethical implications, nearly 30% of faculty members were unaware of AI's prevalence in academic work.

This raises a dilemma for universities: they must either prohibit and penalize AI usage—potentially revising plagiarism policies (Alqahtani and Wafula, 2024; Chan, 2023; Hughes and Roblyer, 2023; Wood, 2023; Yau and Chan, 2023)—or integrate AI into curricula while promoting alternative learning strategies and faculty training. As (Gallent-Torres et al. 2023) suggest, students frequently exposed to AI-related fraud and malpractice discussions may develop generalized behaviors that lead to unethical academic practices. Hence, it is crucial that teachers receive training in ethical and privacy aspects related to the use of AI, ensuring responsible and respectful use of students' rights (Kasneci et al., 2023).

Nevertheless, AI's potential in HE extends beyond concerns over academic dishonesty. AI can enhance learning by personalizing assessments, identifying specific areas for improvement, and adapting content and activities to students' needs (González-González, 2023). AI-driven personalization aligns with the broader objective of providing high-quality, inclusive, and equitable education (Monika Singh et al., 2025).

In favor of the use of AI, a new step in the “normalization” of the AI process is being developed with the publication of the AI Assessment Scale (AIAS) (Perkins et al., 2024). This scale provides a new perspective to think of AI as a tool not to be hidden but rather used in the interest of transparency in the educational field (Caldeiro and Odetti, 2024). The use and philosophy of this tool clearly affects the role of the teacher and the students, and that transparency takes us necessarily to the evaluation of the AI itself as HE institutions around the world.

The emergence of AI tools in education presents a significant challenge for educational institutions, particularly universities: whether to restrict their use or to promote informed and ethical integration into teaching and learning processes. In line with authors such as (Alqahtani and Wafula 2024), (Chan 2023), and (Hughes and Roblyer 2023), we argue that banning such technologies may hinder rather than support educational development. Instead, integrating AI into the classroom, supported by appropriate training for both students and educators, offers a more constructive approach. This also requires a re-evaluation of academic integrity policies, including those concerning plagiarism and originality. As highlighted by (Kasneci et al. 2023), it is essential that educators receive specific training on ethical considerations and data privacy issues related to AI use, ensuring practices that are both responsible and respectful of students' rights.

(Navarrete and Manzanilla 2023) argue that adaptive learning platforms use AI algorithms to assess student performance and adjust pedagogical resources promoting more effective and personalized learning. It is true that there is a paradigm of uncertainty regarding the use of AI, and the integration of these tools in education faces several barriers to their full acceptance in educational institutions (Macías et al., 2024). Likewise, (Tomalá et al. 2023) state that AI has shown significant advances that allow its implementation in educational systems to improve teaching and learning. AI is also a potentially transformative tool capable of creating interactive learning environments that stimulate the active participation of students. Likewise, (Cabrera 2024) highlights the potential these tools provide in transforming the context of assessment in education, due to the detailed analysis provided on students' performance. Using traditional methods, the identification of such patterns and trends could obviously go unnoticed, however using AI tools can aid to perform more accurate assessment and tailored to the competencies or skills of each student.

Building upon this broader potential of AI in education, lesson planning has emerged as a particularly promising area where such technologies can directly assist teachers. Lesson planning remains a cornerstone of effective teaching, functioning as a structured guide for achieving specific learning outcomes. A sound lesson plan typically aligns instructional objectives, materials, classroom activities, and assessment strategies within broader curricular frameworks. Yet, despite its centrality, many educators—both in local and international contexts—face persistent challenges in producing coherent and pedagogically sound plans. These difficulties often include unclear objectives, unbalanced assessment loads, and ineffective instructional language. Such struggles are compounded by the inherent complexity of addressing diverse student needs, integrating culturally responsive pedagogy, and navigating high-stakes testing environments (Loh and Liew, 2016).

In this scenario, artificial intelligence emerges as a promising support tool. AI applications have shown potential to assist teachers in generating adaptable lesson content, fostering instructional creativity, and personalizing materials for varied learner profiles (Yeh, 2024; Octavio et al., 2024). However, the pedagogical effectiveness of these tools depends not merely on their availability but on the teacher's capacity to guide them through well-crafted prompts. As (Karpouzis 2024) notes, prompt engineering has become a core skill—essential to translating AI's computational power into meaningful educational practices. Consequently, integrating AI into lesson planning is not a replacement for teacher expertise but rather an invitation to reconceptualize instructional design through a lens of human-AI collaboration, supported by targeted professional development.

### 2.3 Mathematics and AI in HE

AI has been used in various disciplines (Crompton and Burke, 2023) such as linguistics (Liang et al., 2021), engineering (Shukla et al., 2019), mathematics (Hwang and Tu, 2021), and medicine (Winkler-Schwartz et al., 2019), primarily for access to inclusive learning study materials, self-assessment, and personalized feedback (Hwang and Tu, 2021). However, practical research, like the one we present in this paper, is needed to delve into the changes that AI generates in the teaching-learning process and student performance in specific contexts.

In mathematics, several AI-driven platforms have been developed, such as Khan Academy's personalized learning platform, MathSpring's problem-solving tutorials, and ALEKS' adaptive assessment and learning tools. Of particular interest in this study are Matthew, an AI tool developed by Adaptical, and MagicSchool which, although not exclusively focused on mathematics, helps educators and educational institutions reduce administrative burdens, enabling more personalized instruction, and fostering individual student growth. These tools have been employed for the activity evaluated in our research, as they are applicable to all educational levels and subjects and allow for the generation of practical content and learning situations aligned with the new Spanish educational law (LOMLOE) (Vilar, 2024).

Personalization of learning is one of the most promising opportunities for AI in education, as through adaptive systems, the content and difficulty of tasks this tool can be adjusted according to the progress of the student, improving learning efficiency and increasing motivation (Bolaño-García and Duarte-Acosta, 2024).

Regarding the impact of AI on academic performance, many studies have identified success factors and challenges in implementing AI in educational settings, particularly online environments. However, there is a lack of critical analysis concerning the direct influence of AI on academic performance (Adewale et al., 2024; Tiwari, 2023; Hashim et al., 2022; Seo et al., 2021; Zhou et al., 2021), and research further underscores AI's growing role in mathematics education (Hwang and Tu, 2021).

(García-Martínez et al. 2023) note that assessment measures not only students' acquired skills but also internal and external factors influencing learning, such as aptitude and motivation. AI's ability to analyze large volumes of student performance data enables educators to identify trends and make data-driven pedagogical decisions (Mera Castillo, 2023; Serrano and Moreno-García, 2024). Furthermore, AI tools can provide immediate feedback, accelerating the learning process and allowing educators to focus on qualitative teaching aspects (Zhai, 2022; Monge-Vera et al., 2024).

Concerning instructional designs, (Caldeiro and Odetti 2024) point out the importance of transparency in the use of generative AI. They further say that some educators find the AI as a great potential to improve the efficiency and quality of their educational proposals, whereas others have doubts about the authenticity of the content generated by AI. It is, therefore, essential to underscore the ethical and pedagogical value. In this respect, the AIAS Scale of (Perkins et al. 2024) provides 5 levels for the use of AI in evaluation instances and the degree of intervention of AI in the educational design and evaluation of the process (Caldeiro and Odetti, 2024). According to this scale designed primarily for use in HE settings copes with the ethical challenges brought about using Gen AI to provide a practical solution in its adaptation to a Gen AI assessment approach. In this way, not only is students' engagement enhanced, but it also ensures its ethical usage and promotes skill development. In scale level 4, students are suggested to use Gen AI to create content as a collaborative and creative tool and compare students' results against AI's. This way learners develop critical thinking and evaluative skills by carrying out their own analysis of the use of Gen AI tools.

AI in education offers transformative potential as far as its implementation is guided by ethical considerations, responsible data usage, and a commitment to enhancing student learning experiences. Research is needed to explore how AI can be effectively integrated into HE while preserving academic integrity and critical thinking skills.

The main aim of this study was to integrate AI as an innovative pedagogical tool in university classrooms, encouraging students to utilize it for generating content related to didactic programming in early childhood mathematics education and evaluating its impact on academic performance compared to traditional methodologies.

## 3 Method

To achieve this objective, we used a sequential explanatory mixed-methods design (Creswell and Creswell, 2018), combining both quantitative and qualitative strategies to gain a comprehensive understanding of the effects of AI use in the planning of learning situations. In the quantitative phase, we employed a quasi-experimental post-test only design with experimental and control group. In the qualitative phase, an in-depth analysis was conducted on participants' responses regarding their experiences and outcomes when using AI for the design of didactic proposals. This analysis provided insights into their perceptions, evaluations, and potential improvements in the implementation of this technology within an educational context. Furthermore, it helped us to complete and explain the quantitative results in greater detail.

### 3.1 Participants

The study included two hundred and thirty eight university students enrolled in the undergraduate program in early childhood education. Out of the total number, 2two hundred and nine were female (87.82%) and twenty nine were male (12.18%). The distribution of participants into study groups was non-random. The sample selection was based on convenience, respecting the composition of intact classrooms to ensure that participants remained within their natural class groups. This strategy allowed for the analysis of the effects of implementing AI in the design of learning situations compared to a conventional method. Table 1 shows the distribution by group and gender.

**Table 1:** Distribution of participants by group and gender.

**Experimental**	***n***	***%***	**Control**	***n***	***%***
Woman	74	89	Woman	135	87
Men	9	11	Men	20	13
Total	83	100	Total	155	100

The experimental group comprised 83 students (34.87%) who used AI tools for the design and adaptation of learning situations in a specific educational context. In contrast, the control group, consisting of one hundred and fifty five students (65.13%), carried out the same process using a traditional methodology without AI support. The primary difference between the two groups lies in the use of this technology as a resource for didactic planning.

### 3.2 Instruments

The instruments used to measure the study variables were as follows:

This *ad hoc* template was designed to assess students' initial competence in designing mathematics learning activities aimed at fostering mathematical thinking in early childhood education. While employed as an initial evaluation task, its structure and requirements were comparable to those of the post-test assignment, as both involved the development of an instructional plan focused on a specific numeracy concept. However, unlike the post-test—which required students to integrate all the content covered throughout the course, including both pre-numeracy and numeracy concepts—this initial activity focused exclusively on an introductory topic presented at the beginning of the program. The task was completed without the use of artificial intelligence tools and served as a comparative baseline for evaluating differences in product quality between the control and experimental groups. Both the initial and post-test tasks were assessed using the same rubric to ensure consistency and comparability.

This template was also designed *ad hoc* to collect information regarding the title, educational stage, grade level, subject area, context, objectives, competencies, content, methodology, and assessment. It was provided to students to guide them in the creation of their mathematics teaching units, incorporating usage guidelines for both AI-based tools and traditional methodological resources. The development and validation of this template involved a panel of three experts in mathematics education and instructional design, who reviewed the instrument for clarity, relevance, and curricular alignment. Based on their feedback, a Content Validity Index (CVI) of 0.89 was obtained, indicating acceptable content validity. A pilot trial was also conducted with a small group of pre-service teachers (*n* =) not included in the main study sample, allowing refinement of language, structure, and clarity of the template before implementation.

Assessment rubric for instructors evaluating students' curricular design. This rubric was likewise developed *ad hoc* for the purposes of the study. The design of mathematical learning situations for early years pupils was assessed using a five-point Likert scale (1 = not appropriate, 5 = highly appropriate) across each component: formulation of objectives, competences, content, methodology, and assessment. It also recorded information on the resources used in the task. For students in the experimental group, the evaluation distinguished the level of alignment in the adaptation of AI-generated content according to four predefined categories (no adaptation required, adaptation required but not applied, poor adaptation, and adequate adaptation), based on subject-specific assessment criteria and current educational legislation. A codebook containing detailed operational definitions for each category was used during the evaluation process (see Annexure 3).

This coding scheme was developed deductively based on a general assessment rubric and iteratively refined through expert review to ensure conceptual clarity and relevance. Two independent raters underwent a five-hour training session using exemplar cases and a codebook to calibrate their interpretations of the four-level adaptation scale. They independently coded a subset of twenty five student responses across six rubric dimensions: Objectives, Competences, Content, Context, Resources, and Assessment. Inter-rater agreement was assessed using Cohen's kappa. The overall kappa coefficient was κ = 0.831, reflecting substantial reliability between raters (Landis and Koch, 1977). Item-specific kappa values also indicated strong consistency: Objectives (κ = 0.728), Competences *(*κ = 0.713), Content (κ = 1.000), Context (κ = 0.839), Resources (κ = 0.729), and Assessment (κ = 0.946). Discrepancies were resolved through a structured consensus process involving rubric review and collaborative interpretation of disagreements. This procedure led to minor refinements in the codebook, enhancing its clarity and applicability across the full dataset. The methodological approach adopted here contributed to the transparency, rigor, and reliability of the qualitative coding process.

Grade records. Provided data on students' academic performance, based on final marks, measured on a Likert-type scale from 0 to 10. These marks reflected all the competences and learning outcomes that students were required to achieve in order to pass the module. These grades were based on a comprehensive rubric aligned with the official learning outcomes and competences defined in the curriculum and were assessed by qualified instructors using standardized criteria across both groups. This approach is supported by prior research indicating that course grades, when derived from structured and criterion-referenced assessments, can serve as valid proxies for academic achievement (e.g., Adarkwah, 2021; Allen, 2005; Andersson, 2025; York et al., 2015). In our case, the grading system incorporated multiple components, including instructional design tasks, theoretical understanding, and practical application, thus reflecting a holistic evaluation of students' performance in relation to the module's objectives. Furthermore, the use of grades allowed for a consistent and ecologically valid comparison between the experimental and control groups, as it captured students' actual performance within the authentic learning environment. The integration of detailed rubrics, expert validation procedures, inter-rater reliability statistics, and triangulation with qualitative data (e.g., analysis of submitted tasks and adaptation strategies) helped mitigate potential bias and strengthened the methodological transparency and validity of our findings.

### 3.3 Procedure

In accordance with the ethical standards of the Declaration of Helsinki, students were informed of the objectives of the study, invited to participate voluntarily, assured of the anonymity and confidentiality of their responses, and their informed consent was obtained and recorded. Following this, participants were assigned to two groups: an experimental group and a control group. The experimental group used AI for the design of their didactic units, while the control group followed a traditional methodology without the use of AI.

In both groups, and at each stage (validation task/posttest), the task (Annexure 1) was conceived as a learning situation applicable to any course within early childhood education, which students were required to specify in their proposal. It was designed to be developed collaboratively in class, in pre-assigned groups, with the explicit purpose of preventing the control group from utilizing AI tools during the process. The activity aimed to integrate and apply the contents covered in the subject “Development of Mathematical Thinking,” including early number concepts, verbal problem-solving, classification, and seriation activities. The task was implemented over several class sessions during the second semester of the previous academic year (2023-2024), within the regular schedule of the aforementioned course. Its primary objective was to assess students' general and specific competencies in designing learning situations that foster the development of mathematical thinking in early childhood education.

Students in the experimental group were allowed to work with one of three open-access and free AI tools (ChatGPT, Mathew, or MagicSchool) according to their preferences and needs. They were required to document the prompts used, to include the AI-generated responses, and highlight in a contrasting color any modifications made, accompanied by a theoretical justification explaining the pedagogical relevance of the adapted content and the improvement of their final proposal. In contrast, students in the control group completed the same task without the integration of AI tools, working exclusively during scheduled class hours and under teacher supervision to ensure that AI was not used in completing the task, thereby preserving the integrity of the group comparison intended for the purposes of our research.

It is important to note that the overall pedagogical approach used throughout the course was consistent across both groups. The theoretical components were delivered in a shared format for all students, focusing on key principles of early mathematical thinking and instructional design. Practical learning was organized through collaborative projects and case studies that encouraged the application of theory to authentic classroom scenarios. The main difference between the groups was the integration of AI: while the control group was restricted to conventional tools and resources, the experimental group received explicit instructions on how to formulate and refine prompts to maximize the relevance and quality of AI-generated outputs.

This emphasis on prompt engineering allowed students to critically evaluate the pedagogical potential of different types of AI input, aligning generated content with learning objectives, and adapting it for classroom use. As such, the experimental design not only measured outcomes, but also aimed to develop students' reflective and metacognitive skills in working with emerging technologies.

Once the intervention was completed, data collection for both groups was conducted through the Blackboard Ultra platform over a period of 2 months.

The qualitative analysis of the planning content was carried out in several phases: first, an exploratory reading of the tasks submitted by the students was conducted; subsequently, emergent categories were generated from the content; and finally, the data were systematically coded.

The qualitative coding of content adaptations focused on evaluating how students used AI to generate content suitable for diverse classroom needs. Coding was conducted deductively, based on a four-level rubric (Annexure 2): Does not require adaptation, requires adaptation but not applied, Poor adaptation, and adequate adaptation. The coding logic was as follows:

- No adaptation required. It was assigned when the content generated by AI reached at least level 4 (Good) in all criteria of the general rubric without further modification.
- Adaptation required but not applied. Used when AI-generated content did not reach level 4 in one or more key criteria, and the student made no adaptations.
- Poor adaptation: Applied when the student attempted to adapt the AI-generated content but the resulting quality remained below level 4. Adaptations were minor, superficial, or lacked didactic relevance.
- Adequate adaptation: assigned when the student effectively modified the AI-generated content to reach at least level 4 in the rubric, ensuring quality, inclusivity, and curricular alignment.

This coding system enabled the linkage between pedagogical quality levels and the effective use (or lack thereof) of AI in instructional design, maintaining consistency between qualitative analysis and the predefined evaluative framework.

As previously explained, the qualitative analysis was conducted by two independent researchers to ensure the validity of the study and the consistency of data interpretation. The coding process was developed deductively from a general assessment rubric and refined through iterative expert review. Both coders received specific training using practical examples and a codebook, which allowed them to align their criteria before beginning the analysis. A subset of student submissions was coded independently, and discrepancies were resolved through a structured consensus process involving the review of rubric criteria and collaborative discussion until agreement was reached.

This methodology helped clarify and strengthen the coding system, ensuring its coherence and applicability across the entire dataset. It also contributed to a deeper understanding of students' performance and the pedagogical quality of the learning situations they designed. The final assessment of academic performance was based on the tasks submitted by students from both groups, allowing for a comparative analysis of the impact of AI use versus non-use on academic outcomes.

In order to illustrate the type of coding carried out, an excerpt of the students' work has been included in Annexure 4, where the analysis can be observed in context. The bolded text in the excerpt highlights the specific adaptations made by the students.

### 3.4 Data analysis

We began the statistical treatment of the data with descriptive analyses that included frequencies, percentages, means, standard deviations, medians, and extreme values related to sociodemographic variables, AI tool usage, content adaptations, and academic performance.

To assess potential associations between AI use and the adaptation of curricular content, we conducted chi-square tests of independence that were conducted, along with residual analysis to delve deeper into the interpretation of the results and identify where significant differences were located. Cramer's *V* calculations helped to determine the strength of the association (Cook and Campbell, 1979, 1986).

Subsequently, we conducted inferential analyses using independent samples t-tests. In cases where statistically significant differences were observed, effect sizes were calculated using Cohen's *d* (Cohen, 1988) to evaluate the magnitude of those differences.

The statistical data processing was conducted using Jamovi software, version 1.6.23, with a significance level of 5% and a confidence interval of 95%.

## 4 Results

### 4.1 Initial diagnostic assessment: baseline comparison between groups

To begin the analysis, an initial assessment was conducted to determine whether the control and experimental groups were comparable in terms of their baseline competencies. Prior to conducting inferential analyses, the assumption of normality was tested using the Shapiro–Wilk test, confirming that the distribution of scores met the requirements for parametric testing in both groups (see Table 2).

**Table 2:** Shapiro–wilk test of normality for initial competencies and final performance by group.

**Group**	**Variable**	***W***	***p***
Experimental	Initial competencies	0.978	0.174
Experimental	Final performance	0.977	0.152
Control	Initial competencies	0.99	0.424
Control	Final performance	0.986	0.129

At the beginning of the course, students were tasked with designing a classification activity as an initial diagnostic assessment, explicitly without the use of artificial intelligence. This activity was intended to evaluate their baseline competencies in developing early childhood education activities aimed at fostering mathematical thinking. As shown in Table 3, the evaluation of these tasks revealed no statistically significant differences between the control and experimental groups, *t* (150) = −0.58, *p* = 0.56, indicating that both groups started the course with a comparable level of competence in this area.

**Table 3:** Comparison of initial competencies between control/experimental groups.

**Group**	***M***	***SD***	***t*(gl.)**	***p***
Experimental	6.57	1.27		
Control	6.67	1.15	−0.58(150)	0.56

### 4.2 Use of artificial intelligence tools

Table 4 presents the distribution of frequencies and percentages of the different types of AI tools used by participants.

**Table 4:** Frequency distribution of AI types.

**Levels**	***fr***	**% of Total**	**Cumulative %**
Chatgpt	49	59.0 %	59.0 %
Mathew	29	34.9 %	94.0 %
Magic school	5	6.0 %	100.0 %

The results indicate that, out of all the AI tools used by the students for their designs of mathematics learning situations for elementary education, ChatGPT was the most frequently used AI tool, accounting for 59.0% of the total responses. This suggests that ChatGPT is the preferred AI tool among participants, likely due to its versatility and widespread adoption in educational and professional settings. Mathew was the second most frequently used AI tool, representing 34.9% of the total responses. Finally, Magic School was the least frequently used AI tool, with only 5.0% of participants selecting it.

### 4.3 Adaptation of content for each curricular component

Table 5 (below) shows the percentage distribution of adaptation levels across various educational components, including objectives, competences, context, knowledge, methodology, resources, and assessment. The adaptation levels are categorized as “No needed”, “Need but not do it”, “Bad adaptation”, “Good adaptation”, providing insight into the extent of modification and implementation across different areas.

**Table 5:** Adaptation levels in educational components: percentage distribution.

**Levels**	**Objectives**	**Competences**	**Context**	**Knowledge**	**Methodology**	**Resources**	**Assessment**
	**% of total**	**% of total**	**% of total**	**% of total**	**% of total**	**% of total**	**% of total**
No needed	34.9	33.7	1.2	1.2	1.2	10.8	0.00
Need but not do it		10.8	22.9	25.3	25.3	19.3	20.5
Bad adaptation	6.0	9.6	63.4	28.9	28.6	24.1	60.2
Good adaptation	45.8	45.8	12.0	44.6	44.6	45.8	19.3

The results indicate varying degrees of adaptation across different educational components. While objectives (45.8%), competences (45.8%), resources (45.8%), knowledge (44.6%), and methodology (44.6%) exhibit the highest levels of successful adaptation, context (63.4%), and assessment (60.2%) show the highest percentages of poor adaptation, highlighting significant challenges in these areas. A notable proportion of participants recognized the need for adaptation but did not implement it, particularly in knowledge (25.3%), methodology (25.3%), and assessment (20.5%), suggesting barriers to execution. Additionally, objectives (34.9%) and competences (33.7%) were most frequently considered as not requiring adaptation. The complete absence of responses in the “No needed” category for assessment (0.00%) suggests a universal recognition of its necessity. These findings emphasize the need for improved strategies and support, particularly in contextual adaptation and assessment, to ensure a more inclusive and effective learning environment.

Table 6 presents the chi-square (χ^2^) test results, analyzing whether differences in adaptation levels across educational components are influenced by the type of AI tool used.

**Table 6:** Chi-square test results: differences in adaptation levels based on AI tool used.

**Curriculars items**	***χ^2^***	***df***	***p***	***ε^2^***
Objectives	45.678	2	< 0.001^*^	0.55704
Competences	41.065	2	< 0.001^*^	0.50079
Context	0.220	2	0.896	0.00268
Knowledge	2.035	2	0.361	0.02482
Methodology	0.396	2	0.820	0.00483
Resources	0.901	2	0.637	0.01098
Assessment	1.801	2	0.406	0.02196

The results indicate that differences in adaptation levels are significantly influenced by the type of AI tool used for Objectives (χ^2^ = 45.678, *p* < 0.001, ε^2^ = 0.55704) and Competences (χ^2^ = 41.065, *p* < 0.001, ε^2^ = 0.50079), with large effect sizes. There is a significant and strong association between the use of AI and the adaptation of content for the curricular elements' “objectives” and “competencies”, in favor of those who use AI. This suggests that the AI tool employed has a notable impact on how educators modify learning objectives and competences. However, for Context (*p* = 0.896), Knowledge (*p* = 0.361), Methodology (*p* = 0.820), Resources (*p* = 0.637), and Assessment (*p* = 0.406), the results are not statistically significant, implying that the choice of AI tool does not lead to meaningful differences in adaptation within these components. These findings highlight that while AI use plays a crucial role in shaping learning objectives and competences, other areas may require additional guidance or structured frameworks to ensure effective adaptation. Table 7 displays the standardized results corresponding to the curricular component 'Objectives'.

**Table 7:** Standardized residuals for the association between type of AI and adaptation of objectives.

**Objectives adaptation level**	**ChatGPT (Residual)**	**Mathew (Residual)**	**Magic school (Residual)**
No adaptation needed	−6.14	+6.70	−0.72
Needs but not do it	−0.32	+0.11	+0.46
Bad adaptation	+1.92	−1.69	−0.58
Good adaptation	+5.18	−5.67	+0.66

Chi-square test revealed a significant association between the type of AI tool used and the level of objectives, χ^2^ (6, *N* = 83) = [valor], *p* < 0.001. The analysis of standardized residuals showed that Mathew was significantly overrepresented in the category “no adaptation needed” (residual = +6.70), while ChatGPT was significantly underrepresented in the same category (residual = −6.14). Furthermore, ChatGPT was strongly associated with the “good adaptation” category (residual = +5.18), whereas Mathew was significantly underrepresented in that same category (residual = −5.67). These residuals indicate that ChatGPT and Mathew have different content adaptation patterns, while the School of Magic does not show significant differences in its adaptation levels. The use of ChatGPT was more frequently linked to effective adaptation of curricular content, while Mathew tended to be associated with lower levels of adaptation or lack of perceived need for adaptation. In Table 8, we can see the standardized results calculated for the curricular component 'competencies'.

**Table 8:** Standardized residuals for the association between type of AI and adaptation of competencies.

**Competency adaptation level**	**ChatGPT (Residual)**	**Mathew (Residual)**	**Magic School (Residual)**
No adaptation needed	−5.44	+6.44	−1.65
Needs but not do it	−0.94	+0.64	+0.68
Bad adaptation	+1.72	−2.18	+0.81
Good adaptation	+4.74	−5.21	+0.66

Chi-square test revealed a significant association between the type of AI tool used and the level of curricular competencies adaptation, χ^2^ (6, *N* = 83) = [valor], *p* < 0.001. The analysis of standardized residuals showed that Mathew was significantly overrepresented in the category “no adaptation needed” (residual = +6.44), while ChatGPT was significantly underrepresented in this same category (residual = −5.44). Furthermore, ChatGPT showed a strong positive association with the “good adaptation” category (residual = +4.74), whereas Mathew was notably underrepresented in that same category (residual = −5.21). These standardized residuals indicate, as in the previous case, that ChatGPT and Mathew have different competency adaptation patterns, while the School of Magic does not show significant differences in its adaptation levels. These results indicate that ChatGPT use was more consistently associated with a higher level of competency adaptation, while Mathew tended to cluster in categories requiring no adaptation or showing lower quality adaptation.

### 4.4 Academic performance and use of AI

Finally, Table 9 presents a comparison of performance scores between the experimental and control groups.

**Table 9:** Performance differences by Group.

**Group**	**Experimental**		**Control**		***t***	***p***	***Cohen's d***
	***M***	***DT***	***M***	***DT***			
Performance	8.03	1.07	7.75	0.75	2.12	0.020	0.320

The results indicate a statistically significant difference in performance between the experimental and control groups (*t* = 2.11, *p* = 0.020). The experimental group achieved a higher mean score (*M* = 8.10, *SD* = 1.07) compared to the control group (*M* = 7.75, *SD* = 0.75) suggesting that the intervention applied in the experimental group positively influenced performance.

However, Cohen's d (0.327) indicates that the effect of these differences is between small and moderate implying that, while the difference is statistically significant, its practical impact may be limited. These findings suggest that the implemented intervention had a positive, but modest, effect on performance, highlighting the need for further exploration of factors that could enhance its effectiveness.

## 5 Discussion and conclusions

The objective of this research was to examine how future teachers of early childhood education utilize AI to create learning situations for their students within the didactics of mathematics course. Specifically, we sought to identify which AI tools they employ, the levels of content adaptation they carry out, and which elements of early years mathematics planning involve the use of AI. Subsequently, an analysis was carried out to determine whether there were differences in the content adaptations made by the students depending on the type of AI tool used, as well as in the outcomes achieved, comparing those who employed artificial intelligence with those who relied on traditional teaching resources.

### 5.1 Uses of AI

Based on the results obtained, it was observed that students prefer to use tools such as ChatGPT rather than specific applications that generate lesson plans or learning situations in accordance with the national educational legislation. This trend may be linked to the popularity and growing use of ChatGPT in educational settings, where its adoption has increased exponentially among both students and educators (Hu, 2023). Factors such as ease of use and the adaptability of technological tools are crucial for their acceptance (Selwyn, 2019). This situation raises the need to reflect on the impact that the predominant use of general AI tools, such as ChatGPT, might have on the training of future teachers. It is particularly relevant in terms of their ability to design learning situations that meet legal and pedagogical standards. It also highlights the need to implement specific training programs to enable students to fully harness the potential of AI as a means to address this issue (Luckin et al., 2016).

To create learning situations, students must develop a series of components that structure and give meaning to them, such as objectives, competencies (based on legislation), content, methodology, resources, and evaluation. Additionally, they must adapt the material to the context provided, which includes student profiles requiring one or more adaptations. As observed, different AI tools offer varying levels of accuracy in providing objectives, competencies, and resources. However, they show less precision in technical aspects such as methodology and evaluation. Moreover, none of the submitted works included proper personalization of student learning for those with difficulties by adapting to the provided context.

This discrepancy may arise because the AI-generated tasks require technical and human knowledge derived from direct experience in implementing learning situations, which is difficult to replicate due to the specificity of the task. Another possibility is that students used overly generic prompts, limiting the AI's ability to develop the detailed specifications expected in such activities.

Nonetheless, students successfully adapted to AI-generated objectives, competencies, content, and resources. However, other components, such as methodology, evaluation, and context adaptation, were not redesigned according to the minimum requirements necessary for classroom implementation. As (Martines Rizo 2013) points out, teachers' knowledge of evaluation tends to be limited, making professional development essential for bringing about change in their practices. This aspect has often been neglected, contributing to the persistence of ineffective assessment practices. Proper training in evaluation techniques and approaches is crucial for improving educational quality and ensuring that assessments fulfill their formative and diagnostic purposes.

In the field of mathematics didactics, assessment is often based on the summative evaluation focused on the results obtained, sometimes overlooking the process or the strategies used by students (Boaler, 2016). This was evident in how students adapted AI-generated proposals, as many of the assessment tools, such as rubrics, were measured dichotomously, depending on whether the student achieved the goal or not.

Regarding context adaptation, an essential action to take for personalizing learning for students with diverse difficulties, it was carried out in a generic and imprecise manner, with simple modifications that did not fully address the needs of the proposed classroom. (Angenscheidt Bidegain and Navarrete Antola 2017) argue that as education moves toward a more inclusive approach, less experienced teachers or those still in training face greater challenges in adapting their practices to the current context. These challenges can hinder the effective implementation of inclusive strategies, as they require in-depth knowledge and specific preparation that many pre-service teachers have not yet fully acquired. Consequently, these educators are more likely to encounter obstacles when attempting to integrate inclusive practices.

### 5.2 Levels of content adaptation by type of AI used

Our data shows significant differences in the way students adapted learning objectives and competencies based on the specific AI tool they employed. Notably, substantial variations emerged between ChatGPT, Mathew, and Magic School, highlighting the distinctive features of each tool in terms of design and functionality.

It is highly interesting to note that, when focusing on the adaptations made by students based on the AI tool used, significant differences emerged regarding the alignment of the objectives and competencies of the learning situations with the specificity of each tool. In particular, substantial variations were identified between ChatGPT, Mathew, and MagicSchool. Mathew, for example, excels in generating learning situations that are closely aligned with the curriculum and current educational legislation. Its design allows for the automatic creation of personalized content that precisely matches educational objectives and competencies. As a result, students who used Mathew found that the generated objectives and competencies were sufficiently aligned with curricular expectations, significantly reducing the need for additional adaptation. Conversely, students working with more general tools like ChatGPT or MagicSchool often needed to make manual adjustments to ensure compliance with curriculum standards, highlighting the differences in the level of alignment and the effort required by the user.

Mathew, for instance, is particularly noteworthy for his ability to generate learning scenarios that are strictly aligned with national curriculum standards and educational legislation. Its design allows it to produce customized content that precisely adheres to the objectives and competencies established in official regulations. Consequently, students using Mathew often find that the generated objectives and competencies already meet curricular expectations, significantly reducing the need for additional modifications. In other words, Mathew enables students to focus on other aspects of the instructional process, as the fundamental components related to objectives and competencies are pre-defined and aligned with official standards.

Another factor contributing to the reduced need for adaptation among students utilizing Mathew is the tool's high level of relevance in selecting objectives and content. Mathew appears to provide exceptionally accurate correspondence between proposed learning situations and educational objectives, leading students to perceive minimal necessity for further adjustments. This precise alignment between generated content and curricular needs stands as a key factor in explaining the observed differences in adaptation efforts compared to tools like ChatGPT and Magic School, where students often found it necessary to make adjustments to ensure alignment with educational objectives and competencies.

In light of the above, we can affirm that while AI tools may offer a valuable foundation for designing learning situations, their effectiveness not only depends on the precision of the tool itself but also on the user's ability to critically interpret and refine the generated results. Tools like Mathew demonstrate that highly specialized AI can reduce the workload associated with curricular alignment, allowing users to focus on methodological and contextual adaptations. However, more general tools require greater professional intervention. In this way, this study highlights the need for robust teacher training that will equip future educators with the analytical skills and pedagogical judgement necessary so that they can effectively use AI tools, compensate for their limitations, and ensure educational quality and compliance with regulations.

### 5.3 Adaptation to context, content, methodology, resources, and assessment

No significant differences were identified among the various AI tools regarding the adaptation of contextual elements, content, instructional methodology, resources, and assessment strategies. Regardless of the AI tool used, students applied similar adaptations in these aspects. However, it is essential to highlight that a lower degree of effectiveness was observed in the contextual adaptation and assessment of AI-generated content. This suggests that while AI tools provide useful proposals, students must apply their technical and pedagogical expertise to refine and adapt them effectively for a real-world implementation.

This process of customization requires specific competencies that students are still in the process of developing, limiting their ability to make fully effective adaptations. As a result, despite AI tools are valuable resources, their use demands a level of proficiency that students have not yet fully acquired. It, therefore, underscores the need for further refinements to make sure that content is appropriately tailored to the educational context and assessment criteria.

Nonetheless, most students successfully adapted instructional methodologies and resource selection for the implementation of the activity. This success may be attributed to the comprehensive teacher training programs that integrate methodological aspects across various didactic approaches, fostering constructivist teaching practices. Such an approach is particularly relevant in early mathematics education. The collected data reinforces the notion that transforming teaching conceptions and practices is a gradual process fraught with challenges. Teacher education does not seek to replace students' initial ideas with those deemed superior by educational research but rather adopts progressive, constructivist approaches that encourage the evolutive development of teaching practices.

This philosophy aligns with the student-centered training model, which prioritizes the gradual and reflective development of competencies and knowledge. Consequently, teacher education is geared toward a transformation process that, although slow, promotes more sustainable and effective changes in educational practices, aligning with the demands of a more constructivist and adaptive teaching paradigm (Rivero et al., 2011).

### 5.4 Critical reflection and investigative learning approach in the use of AI

It is essential to emphasize that all student adaptations involved a rigorous process of critical reflection, regardless of the final outcome. This process required students to critically evaluate which content, resources, and methodologies were more suitable for the designed learning situation while identifying areas that required modifications for successful implementation. Consequently, the task was taken on a research nature, demanding continuous assessment and refinement of educational components, irrespective of the AI tool utilized.

This investigative approach fosters critical thinking, enabling students to question and select the most relevant and appropriate information for their specific contexts (Betancourth Zambrano, 2015). Furthermore, this process encourages an in-depth and deliberate analysis of pedagogical decisions, strengthening the critical thinking skills essential for effective teaching. It is crucial for training future educators to develop these competencies through reflection and inquiry, as it helps them adapt their practices to diverse learning needs while cultivating a more conscious and evidence-based approach to their professional responsibilities.

### 5.5 Impact of AI on academic performance

The findings of this study reveal statistically significant differences between students who used AI in the development of learning situations and those who did not. Students who incorporated AI tools into their analytical and adaptation processes demonstrated slightly higher academic performance compared to their peers who did not utilize such technologies, although the effect size (Cohen's d ≈ 0.33) suggests a small to moderate impact, limiting practical implications.

The results align with existing literature suggesting that AI tool implementation can be a beneficial strategy to enhance academic outcomes. (Essel et al. 2022), for instance, examined the impact of virtual assistants in higher education in Ghana, finding that students interacting with chatbots outperformed those in traditional instruction. However, given this study's posttest-only design, lack of random group assignment, and use of unvalidated rubrics, these results should be interpreted cautiously.

These findings point to AI's promise as an educational aid, reinforcing the need to explicitly link empirical outcomes with established theories. Students must be equipped with strong analytical skills to critically assess AI-generated content. Nevertheless, claims about AI's effectiveness should be tempered due to methodological constraints, and the evidence does not support broad generalizations about its overall impact on learning.

AI may assist with repetitive tasks and shift focus toward deeper learning processes. Yet, despite statistical significance, the effect's limited size and absence of a pretest reduce the strength of conclusions. Future research should adopt more rigorous designs and clearly connect results with theoretical frameworks to clarify AI's educational value.

The data show no major performance differences based on the specific AI tool used. All tools require users to apply interpretive and evaluative skills, indicating that these competencies—not tool type—are central to academic success. Still, results should not be overextended beyond what the evidence allows.

This study explored how pre-service early childhood and elementary teachers use AI to design learning scenarios in the undergraduate course of didactics of mathematics. Students favored general tools like ChatGPT over specialized applications aligned with national curricula. This suggests accessibility influences preferences, though it raises questions about pedagogical adequacy and legal compliance.

While AI supports drafting objectives and competencies, it lacks precision in methods, assessment, and adaptation for students with special needs. This contrasts with literature on AI's personalization potential, revealing that complex educational tasks still require expert human judgment. Limitations should be more explicitly addressed, ideally in a dedicated section.

AI can offer personalized learning opportunities (Mera Castillo, 2023; Zhai, 2022), yet its integration must respect pedagogical standards. This study reaffirms the indispensable role of teachers in guiding students, especially given the significant limitations of the current research design.

### 5.6 AI as an educational complement

These findings suggest that AI can serve as a valuable complement, particularly in routine or lower-complexity tasks, enabling students to focus on developing their analytical and adaptive skills. By leveraging AI to automate mechanical aspects of academic work, students can allocate greater cognitive resources to evaluating and refining content quality.

In conclusion, while AI tools may offer an initial framework for the design of learning activities, their effectiveness largely depends on the user's ability to critically interpret and adapt the generated outputs. Tools such as Mathew exemplify how domain-specific AI can streamline certain instructional design processes, whereas more general tools often necessitate greater pedagogical mediation.

This study highlights the importance of equipping future educators with the analytical and pedagogical competencies required to engage meaningfully with AI technologies, harness their potential benefits, and uphold educational standards. Accordingly, the findings should be interpreted as exploratory and serve as a foundation for further empirical research in this area.

Finally, future directions may include expanding the implementation of this approach to additional courses, conducting ongoing evaluations of emerging learning modalities, and fostering students' critical capacities, particularly in content adaptation and personalization, as key factors in optimizing the role of AI in competency-based higher education.

### 5.7 Limitations and future directions

Despite the valuable findings obtained, this study presents several limitations that should be considered to guide future research. Firstly, the use of final course grades as the sole measure of academic performance limits the objectivity and generalizability of the results, as these grades, although based on detailed rubrics, do not constitute a standardized or independent indicator of learning. To overcome this limitation, it is recommended that future studies incorporate externally validated assessment instruments that allow for a more precise and comparative measurement of the impact of AI on student performance.

Secondly, the methodological design based on a post-test-only scheme without randomized group allocation, although justified by logistical and ethical considerations, restricts the ability to establish strong causal relationships. Future research should consider more rigorous experimental designs, such as randomized controlled trials, to enhance internal validity.

Additionally, the sample was selected for convenience, which limits the representativeness of the findings; therefore, it would be desirable to expand the sample to include different university contexts and disciplines to improve generalizability.

Another important limitation is that the learning situations designed were not implemented in real early childhood education classrooms, which prevents the evaluation of their applicability and effectiveness in authentic settings. Future studies should include implementation and observation phases in real-world environments to validate the pedagogical relevance of AI-generated proposals.

Moreover, students demonstrated limited ability to adequately adapt AI-generated content in key areas such as assessment, methodology, and contextual personalization, highlighting the need to strengthen teacher training in analytical, ethical, and pedagogical competencies for the critical use of these technologies.

Finally, although triangulation and peer review strategies were employed to strengthen the qualitative coding process, the absence of a quantitative measurement of formal double coding may have affected the reliability of the analysis. Consequently, it is recommended that future studies incorporate more robust inter-coder validation procedures. Addressing these limitations will enable progress toward a deeper, more rigorous, and contextually grounded understanding of the role of AI in teacher education and its impact on educational quality.

Then, as future directions, it is proposed to overcome the limitations of this exploratory study through practical actions such as extending this experience to other courses, the continuous evaluation of these new forms of learning, and the strengthening of students' critical skills, particularly in the adaptation and personalization of content. These areas are considered key to maximizing the positive impact of AI on university-level, competency-based learning.

One of the main limitations of this study lies in the use of final course grades as the primary measure of academic performance. Although these grades were based on detailed rubrics aligned with the official learning outcomes and competences of the module, and were applied consistently by qualified teaching staff, we acknowledge that they do not constitute an independent or standardized measure of student achievement.

This choice was made due to logistical constraints and the need to assess performance within an authentic learning environment. Nevertheless, future research would benefit from the inclusion of externally validated instruments that allow for a more objective and generalizable evaluation of the impact of pedagogical interventions, particularly in contexts involving the integration of emerging technologies such as artificial intelligence.

Another limitation of the study is that the designed learning situations were not implemented in actual early childhood education classrooms. As a result, it is not possible to determine whether the adaptations made by the pre-service teachers, based on AI-generated materials, effectively respond to the real needs and dynamics of a preschool classroom. Furthermore, without classroom implementation, it remains unclear whether these proposals meet the legal and curricular requirements established for early childhood education in practice.

These limitations, while relevant, were addressed through practical safeguards to preserve the internal consistency and pedagogical coherence of the study.

## Data Availability

The raw data supporting the conclusions of this article will be made available by the authors, without undue reservation.
