# Supplementary material for: Evaluating the use of AI in the design of learning situations by university students of early childhood education
Source: Front Psychol. 2025 Sep 24;16:1604414. doi: 10.3389/fpsyg.2025.1604414 (PMC12533272; doi:10.3389/fpsyg.2025.1604414)
Supplement: Supplementary file 1 [file Supplementary_file_1.docx]

**Appendix**

**Annex 1**

**Learning Situations Template**

| TITLE | Stage and grade | Work areas |
| --- | --- | --- |
|  |  |  |
| CONTEXT | | |
| CURRICULAR FOUNDATION | | |
| Objective(s) contributed to | | |
| Specific competency | Assessment criteria | Content (core knowledge) |
| Methodology | | |
| Methods (styles, strategies, techniques) | Student organization | Schedule (sessions and course timing) |
| Space organization | Materials and resources |  |
| Attention to individual differences | | |
| STUDENT ASSESSMENT PROCESS | | |
| Achievement indicators | Techniques and tools | Timing |
|  | Agent | Criteria (I am able to...) |
| Connection with school projects | Supplementary activities |  |
| ACTIVITY AND TASK PLANNING | | |
|  |  |  |
|  |  |  |
|  |  |  |

**Annex 2**

Curriculum Design Assessment Rubric (AI-Supported Learning Situation)

General Criteria – 5-Point Likert Scale

| Criteria | 1 - Very Poor | 2 - Poor | 3 - Acceptable | 4 - Good | 5 - Excellent |
| --- | --- | --- | --- | --- | --- |
| Learning Objectives | Objectives are missing or irrelevant. | Objectives are unclear or poorly written. | Objectives are appropriate but vague or general. | Objectives are clear, specific, and coherent. | Objectives are precisely formulated, highly specific, and well aligned with competencies and content. |
| Competency Definition | No competencies are identified. | Competencies are inappropriate or poorly formulated. | Competencies are included but underdeveloped. | Competencies are well chosen and clearly stated. | Competencies are excellently formulated, relevant, and well aligned with curricular goals. |
| Content Selection and Organization | Content is irrelevant or disconnected from objectives. | Content is minimal or poorly structured. | Content is appropriate but could be better organized. | Content is well selected and logically sequenced. | Content is highly relevant, well-structured, and contextually appropriate. |
| Context | The explanation lacks any reference to student needs or contextual factors. No support measures are mentioned. | Some recognition of the need for support, but no concrete measures are provided. Response is vague or generic, with no clear connection to student needs or objectives. | The explanation identifies student needs and includes some support measures, but they are general or only partially aligned with learning objectives. | Clear identification of student needs and context. Describes appropriate support measures that are mostly concrete and aligned with objectives. | Thorough understanding of context and student needs. Provides specific and well-justified support measures that are clearly adapted to the learners’ needs and aligned with the intended learning goals. |
| Didactic Methodology | No clear methodology is described. | Methodology is inappropriate for the educational context. | Methodology is acceptable but lacks innovation. | Methodology is clearly defined and appropriate. | Methodology is well-grounded, creative, participatory, and well justified. |
| Assessment Design | No assessment strategy is described. | Assessment is unclear or misaligned. | Assessment is adequate but generic. | Assessment is well-designed and aligned with learning objectives. | Assessment is comprehensive, coherent, and uses varied, relevant tools. |

Annex 3 Qualitative Coding Framework for Student Work Analysis

| Level | Description |
| --- | --- |
| Does not require adaptation | The content generated by the AI meets all rubric criteria at Level 4 (Good) or higher without any need for modification. The proposal demonstrates quality, curricular coherence, and pedagogical soundness. |
| Requires adaptation but not applied | The AI-generated content falls below Level 4 in one or more key criteria of the general rubric. No adaptations or improvements were made by the student, and the final product retains the original weaknesses. |
| Poor adaptation | The student attempted to modify the AI-generated content, but the final result still falls below Level 4 in the general rubric. Adaptations are minimal, superficial, or lack pedagogical justification. |
| Adequate adaptation | The student successfully revised the AI-generated content, resulting in a final version that reaches at least Level 4 in all key criteria. Adaptations improve quality, inclusivity, and curricular alignment. |

Annex 4 Examples of AI-Generated Content and Student Adaptations by Curriculum Component

| Component | No adaptation required | Adaptation required but not applied | Poor adaptation | Adequate adaptation |
| --- | --- | --- | --- | --- |
| Objectives | AI generated:  To become aware of one’s own body and that of others, as well as its possibilities for action, and to learn to respect differences. To develop an accurate and positive self-image.  (Royal Decree 157/2022) | AI suggested: “To begin developing logical and mathematical thinking skills..” Vague and not measurable. Student kept it unchanged. | Student rewrote: To begin developing logical and mathematical thinking skills, **as well as reading and writing, and movement, gesture, and rhythm.** | Student revised to:  To develop basic technological competencies and begin using them for learning, fostering a critical attitude toward how technology functions and the messages it conveys and produces. **(Royal Decree 157/2022)** |
| Methodology | **Learning styles:** ⚬ **Visual:** Learning by seeing, through the use of images and photographs, for example, in classification activities. ⚬ **Auditory:** Learning by listening, through activities such as reading aloud and following verbal instructions. ⚬ **Kinesthetic:** Learning by doing, through hands-on tasks and practical knowledge.  Students will work in pairs and small groups. Each group will receive a link to an interactive digital presentation. The classroom will be organized around collaborative workstations, with tables arranged in groups of five. In addition, there will be designated areas for assemblies, digital technologies, and a library space. | AI suggested:  To develop this learning situation, we will use various (active) methodologies depending on the nature of the activities and the specific learning goals at each moment. The student will always be the protagonist of their own learning process, while the teacher will act as a guide, supporting and directing them according to their needs and interests.  Thus, a play-based methodology will be used, as play holds great importance at this stage of development. | The working methodology for this proposal will be based on experiences, structured activities, and play. All of this will take place within an environment of affection and trust, with the aim of fostering students’ self-esteem and social integration, thus making the teaching and learning process as engaging and effective as possible.  **Therefore, this refers, on the one hand, to an active methodology, which promotes student participation through meaningful experiences and real-world questions, and on the other hand, to a cooperative methodology, encouraging collaborative learning among peers.** | The learning situation is designed to combine various active and functional methodologies, based on students' interests. It promotes different forms of groupings, allowing students to work collaboratively **in the creative resolution of the proposed problem.**  **Additionally, the design follows the principles of Universal Design for Learning (UDL), meaning that all activities are scaffolded to ensure that every student can actively participate in the learning process.**  **The following methodological strategies will be used:**  **Experiential learning**  **Collaborative work**  **Individualized work**  **Daily routine technique (class assembly)** |
| Context | We will begin from the principles of Universal Design for Learning (UDL), providing a teaching and learning process that is appropriate and accessible for all students.  In our case, we have a student with Autism Spectrum Disorder (ASD). For this reason, sessions will be clearly structured, and the student will be informed in advance about the day’s schedule and the types of activities to be carried out.  At their desk, the student will have a detailed visual timetable, indicating the subject, the teacher leading the session, any specialists who may join the class, and the different learning spaces to be used throughout the day.  Content will be adapted to the student’s learning level and presented in multiple formats. To achieve this, we will use pictograms, videos, gestural communication, and hands-on activities, while providing ongoing motivation and encouragement.  The student will be seated in a strategic location that compensates for their difficulties—close to the teacher and relevant materials—and the classroom will be adapted to meet their specific needs. | A series of general measures will be implemented to prevent potential difficulties and, where necessary, to help overcome them:  Flexible grouping arrangements  Adaptable schedules and learning spaces to accommodate the needs of various group configurations | The proposed activities will be designed following the principles of Universal Design for Learning (UDL), with the goal of ensuring accessibility and participation for all students.  **To this end, we will provide multiple means of accessing information (visual, auditory, and tactile), using visual cues (such as pictograms, images, or real objects) and auditory prompts, engaging as many senses as possible.** | **Attention to diversity is regulated by Decree 23/2023, of March 22, issued by the Regional Council of Government,** which governs the educational response to individual differences among students in the Community of Madrid.  In our classroom, we have a student diagnosed with **Attention Deficit Hyperactivity Disorder (ADHD)**. The following measures will be implemented:   - **Assigning tasks that involve physical movement** - **Allowing extra time to complete tasks when needed** - **Ensuring comprehension of instructions before starting any activity** - Assigning a **peer tutor** to help the student regain focus when distracted   **In addition, we will apply reinforcement and enrichment measures for students who progress at slower or faster paces. Finally, the principles of Universal Design for Learning (UDL) will be applied throughout.** |
| Assessment |  | The AI provides the following assessment criteria:   - I am able to compare sizes. - I am able to classify objects. - I am able to count. - I am able to recognize basic shapes. | To evaluate this learning situation, we will take into account a range of techniques and tools, detailed below:  – Techniques, Instruments, and Evaluation Agents:  ▪ Checklists and records to gather information on student learning:  ● Record of habits, attitudes, and personal autonomy  ● **Contextualized observation records**  **● Daily log**  **● Anecdotal record** | Techniques and Instruments  Timing  Agent  “I Can” Criteria  I can correctly identify the category of an object.  I can correctly match characteristic cards with object images.  I can correctly order objects based on the characteristic being compared.  I can correctly guess the name of an object based on the characteristics observed.  I actively participate in the activity and group discussions.  I can correctly place the crocodile’s mouths.  **Techniques:**  **Direct observation**  **Self-assessment**  **Instrument:**  **An evaluation rubric will be used.**  **The following items will be assessed:**  **Active participation in activities**  **Collaboration in the development of activities**  **Compliance with the rules of each activity**  **Acquisition of the contents and concepts of the activities**  **Activity: “Which Object Is It?”**  **This activity will be carried out during the unit on ordering and sequencing.**  **Activity: “The Crocodile’s Mouth”**  **This activity will be introduced alongside the unit on basic quantifiers.**  **Evaluation Agents:**  **The teacher will act as the evaluator in the “Which Object Is It?” activity, along with student self-assessment.**  **The teacher will also be the evaluator for the crocodile activity and will explain its development.**  **Additional “I Can” Criteria:**  **I can put objects in order based on the characteristic being compared.**  **I can distinguish between “greater than,” “less than,” and “equal to” for the objects presented.**  **I can identify which numbers are greater or smaller.** |
